# Impact of template-based synoptic reporting on completeness of surgical pathology reports

**DOI:** 10.1007/s00428-023-03533-6

**Published:** 2023-04-05

**Authors:** Nicole Schaad, Sabina Berezowska, Aurel Perren, Ekkehard Hewer

**Affiliations:** 1 Institute of Tissue Medicine and Pathology, University of Bern, Bern, Switzerland; 2 Present Address: Department of Laboratory Medicine and Pathology, Institute of Pathology, Lausanne University Hospital, University of Lausanne, Rue du Bugnon 25, 1011 Lausanne, Switzerland

**Keywords:** Synoptic reporting, Colon cancer, Lung cancer, Structured reporting

## Abstract

Synoptic reporting increases completeness and standardization of surgical pathology reports and thereby contributes to an increased quality of clinical cancer care. Nevertheless, its widespread practical implementation remains a challenge, which is in part related to the effort required for setup and maintenance of database structures. This prompted us to assess the effect of a simple template-based, database-free system for synoptic reporting on completeness of surgical pathology reports. For this purpose, we analyzed 200 synoptic reports (100 colon and 100 lung cancer resections each) for completeness as required by the pertinent College of American Pathologists (CAP) protocols and compared these to a control dataset of 200 narrative reports. Introduction of template-based synoptic reporting resulted in improved completeness (98% of mandatory data elements) as compared to narrative reports (77%). Narrative reports showed a high degree of completeness for data elements covered by previously existing dictation templates. In conclusion, template-based synoptic reporting without underlying database structure can be a useful transitory phase in the implementation of synoptic reporting. It can result in a similar degree of completeness as reported in the literature for database solutions and provides other benefits of synoptic reporting while facilitating its implementation.

## Introduction

Synoptic reporting contributes to the quality of surgical pathology reports for cancer specimens by increasing completeness and standardization [1, 2]. The College of American Pathologists (CAP) defines synoptic reporting by (1) completeness in terms of adherence to a checklist of required data elements (RDE), and (2) a laboratory value-like paired format consisting of the RDE and the matching response with (3) different RDE presented in separate lines [3]. Comprehensive sets of cancer protocols are published by the CAP and by the International Collaboration on Cancer Reporting (ICCR) [4]. Synoptic reporting—according to the above definition—is an important step on the way to higher levels of data structuring, which include use of discrete data fields and the link to underlying ontologies such as SNOMED-CT [5].

As CAP-accredited pathology laboratories are required to use the CAP cancer protocols, a number of commercially available solutions have emerged, which provide suitable database structures, maintenance of protocols and interfaces to local laboratory information systems. Outside the USA or in languages other than English—with the noteworthy exception of the nation-wide database provided by PALGA in the Netherlands—a significant burden is upon pathology departments in order to implement synoptic reporting and to continuously update protocols. Standardized cancer reporting protocols have been made available by the ICCR in French, Spanish, and Portuguese [6]. Regarding synoptic reporting in German, there have been individual efforts to assure usage of standardized vocabulary [7], but general database solutions are not yet widely applicable.

Based on these considerations, we attempted to facilitate implementation of synoptic reporting at our institution by separating issues related to the actual content and its formatting from those related to the setup of a database. For this purpose, we took advantage of the autotext features of our laboratory information system in order to create templates in synoptic format (matching the corresponding CAP protocols and translated to German; for certain protocols, also to French). The CAP protocols then served as checklists for the pathologists either for dictating or for entering the responses themselves. This system was intended as a transitory solution, the experiences from which would then instruct the design and implementation of an underlying database.

Here, we assess the effect of this template-based, database-free synoptic reporting system on completeness of surgical pathology reports.

## Methods

The synoptic reporting template for lung cancer was introduced in July 2016 (Version 3.4.0.0 of the CAP protocol) and underwent a major update (Version 4.0.0.2) in April 2018. The colon cancer template (Version 4.0.0.1 of the CAP protocol) was implemented in November 2017. After implementation of either protocol, all pertinent reports were rendered in synoptic format. We analyzed 100 consecutive lung cancer and colon cancer synoptic reports each. Reports from the first 3 months after implementation of either protocol were excluded in order to reduce potential effects related to the pathologists’ or transcriptionists’ learning curve or early minor modifications of the templates. A total of 100 consecutive narrative reports for lung and colon cancer each served as control. In order to minimize confounding effects, we chose these from the period immediately before implementation of the respective synoptic protocol. Carcinomas of the rectum were excluded from evaluation of both the synoptic and the narrative reports. All cases from the two study periods had been reported in German.

Reports were reviewed and each data element was classified as “present,” “missing,” or “not applicable.” While the CAP protocols subsume lymphatic and vascular invasion under the umbrella term of lymphovascular invasion, we chose to report them as separate items in accordance with the TNM classification. Therefore, they were also analyzed separately for completeness. Within our institutional policy for implementation of synoptic reporting, we decided to include all mandatory data elements as per the respective CAP protocols, whereas the inclusion of optional data elements was at the discretion of each of the department’s subspecialty groups. Since “Treatment effect” became a mandatory data element only with the introduction of version 4.0.0.0 of each of the protocols, it was excluded from analysis for the purpose of the present study.

Completeness for each data element was defined as 100% minus the percentage of missing elements. Non-applicable data elements as per the CAP protocols—which usually had not been specifically mentioned in narrative reports—were excluded from analysis.

The templates had been tailored to use formatting features available in our laboratory information system, particularly bold font and italics as well as empty lines. On the other hand, vertical alignment of responses was not feasible, as the laboratory information system did not provide a suitable way of using tables or tabulator stops within templates (Tables 1 and 2). Each template contained a heading that specified the synoptic nature of the report as well as the name of the protocol. This heading was followed by the TNM formula, as we intended it to be as easily retrievable as possible. At the end of each synoptic template, the version of the protocol and its source were mentioned. This final line also served to separate the synoptic report from possible additional narrative elements.

**Table 1:** English translation of parts of the synoptic template for colon cancer

Synoptic report (primary carcinoma of the colon, resection) TNM Classification (UICC, 8th Ed., 2017): **pT pN L V Pn G R**
Procedure:
Tumor site:
Tumor size (greatest dimension):
Macroscopic tumor perforation:
Histologic type:
Histologic grade:
Tumor extension:
Lymphatic invasion:
Vascular invasion:
Perineural invasion:
[…]
Additional pathologic findings: ### if applicable; otherwise, delete the line
*(End of the synoptic report, CAP Protocol Version 4.0.0.1)*
### include separate template for MMR status if applicable

Templates were tailored to the functionalities of our laboratory information system: bold font, italics, and empty lines are used for structuring the text (and are correctly rendered in the printed or PDF version of the report). Conversely, tables or tabulator spaces are not available so that text cannot be aligned vertically. Multiple hash (#) signs can be used for comments as the system is programmed to prevent reports from being signed out until the hash signs have been removed. Versioning of both TNM classification and synoptic protocol is included in the template in order to facilitate later use of the data

**Table 2:** English translation of parts of an actual narrative report (upper part) from the study and a synoptic version created from its content (lower part) for a colon cancer resection

Right hemicolectomy:
Mucinous adenocarcinoma of the proximal ascending colon, greatest tumor diameter of 6.9 cm, with infiltration of the pericolonic adipose tissue
[…]
Lymphatic invasion identified. Vascular invasion not identified. No perineural invasion
Oral (ileal) and aboral (colonic) margins free of tumor
[…]
Procedure: right hemicolectomy
Tumor site: right (ascending) colon
Tumor size (greatest dimension): 6.9 cm
Macroscopic tumor perforation: [*missing in the narrative report*]
Histologic type: mucinous adenocarcinoma
Tumor extension: tumor invades through the muscularis propria into pericolorectal tissue
[…]
Lymphatic invasion: present
Vascular invasion: not identified
Perineural invasion: not identified
Proximal margin: uninvolved by invasive carcinoma
Distal margin: uninvolved by invasive carcinoma
[…]

One required data element (macroscopic tumor perforation) is missing in the narrative report, and terminology is more variable and arguably prone for misinterpretation. For example, the positive response for lymphatic invasion and the negative response for vascular invasion differ only by the word “not,” the accidental omission of which would invert the meaning. The synoptic report spans over more lines, but specific pieces of information can arguably be retrieved more easily

**Fig. 1:**
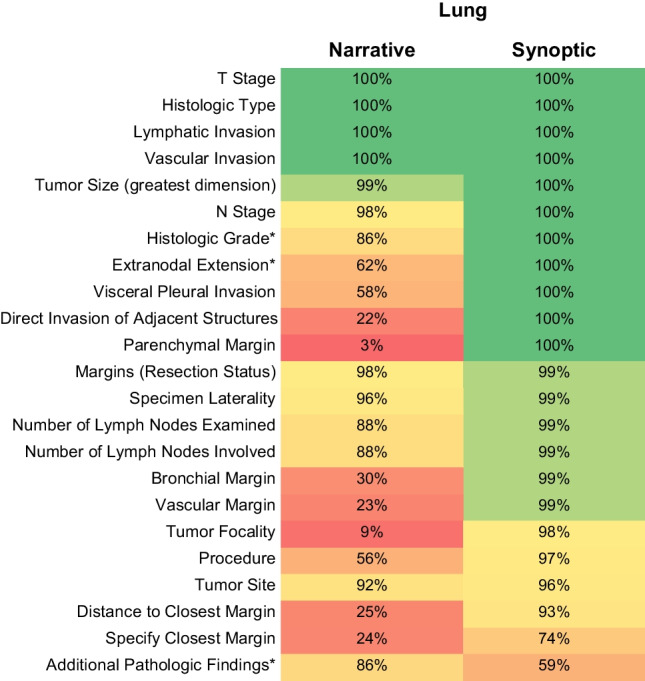
Completeness of data elements for lung cancer resection specimens through 100 consecutive narrative and synoptic reports, respectively. Asterisks (*) indicate optional data elements as per the CAP protocol

Multiple hash (#) signs were used to mark comments as well as optional or conditional elements. These provided a way to remind users of information that we felt might otherwise be forgotten. An English translation of parts of a protocol is given in Fig. 1. Pathologists were also provided with a PDF version of the original CAP protocols, to which we had added comments for internal use and suggested translations of English terminology.

## Results

### General findings

When analyzing the 100 consecutive reports for each cancer type, we found that the synoptic templates had been used for each applicable resection specimen, corresponding to 100% adherence to the synoptic format. Occasional minor terminological variations were identified (such as “no” rather than “not identified” as a response). When we considered these as unequivocally understandable for clinical colleagues, we counted them as valid responses. Synoptic reports typically spanned more lines than narrative reports, but, at least, subjectively, the pertinent pieces of information were more easily retrievable from synoptic reports as compared to narrative reports. Given that any remaining hash (#) signs from an internal note would block electronic signature, no such erroneously remaining notes appeared in the reports. We did not observe any other recurrent formatting issues among synoptic reports either.

### Lung cancer

For lung cancer, overall completeness rate was 96% for synoptic as compared to 67% for narrative reports. For mandatory data elements, completeness was 98% among synoptic and 65% among narrative reports. Detailed results are shown in Fig. 1. Of note, the only (optional) element that was more frequently reported with the narrative format was “Additional pathologic findings.” Narrative reports showed the highest rate of completeness (≥ 98%) for the histological type and all elements covered by a previously existing template for the TNM formula (i.e., T and N stages, lymphatic invasion, vascular invasion, and resection status), with the exception of histologic grade that was missing in 14% of cases. Among synoptic reports, the specification of the closest margin was the only mandatory element that was reported in less than 90% of cases, possibly due to how the pertinent remark was given in the template.

### Colon cancer

**Fig. 2:**
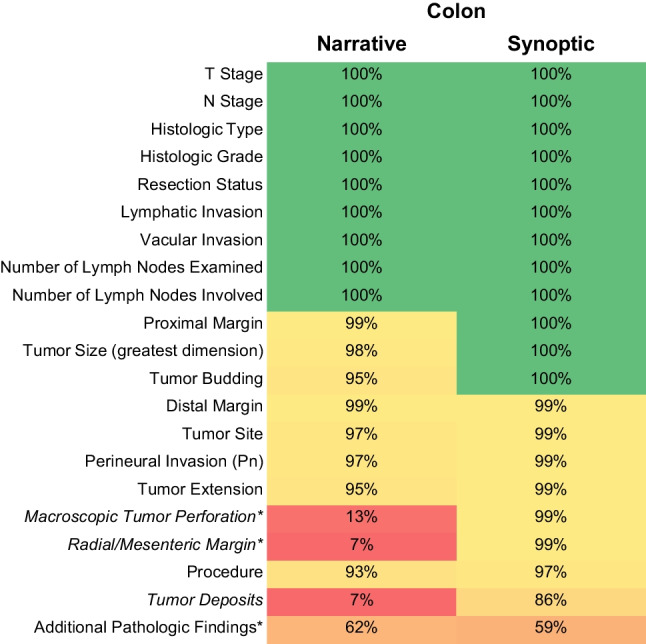
Completeness of data elements for colon cancer resection specimens through 100 consecutive narrative and synoptic reports, respectively. *Italics* indicate data elements not present in the previous narrative dictation template. Asterisks (*) indicate optional data elements as per the CAP protocol

Overall completeness was 97% for synoptic reports as compared to 93% for narrative reports (Fig. 2). In contrast to lung cancer specimens, a dictation template had been systematically used for colon cancer resections before implementation of synoptic reporting.

All elements covered by this dictation template were reported in > 90% of cases. In contrast, three data elements not covered by the dictation template were only infrequently reported in narrative reports. These included tumor deposits, which represent a mandatory data element as per the CAP protocol. Consequently, the most pronounced increase in reporting frequency in synoptic reports was seen for these three data elements. Again, the only data element with a (slight) decrease in completeness in synoptic reports was “Additional pathologic findings.”

## Discussion

In the present study, we have assessed the effect of our template-based approach on completeness of colon and lung cancer resection reports and found very high rates of 98% completeness across all mandatory data elements using synoptic reports. To our knowledge, this is the first study to assess completeness of reports associated with synoptic format but without concurrent implementation of some kind of database structure. These values in terms of completeness are in a range at least similar as those reported for database solutions [8–10], specifically above 95% and, in many cases, reaching 100%. Equally in accordance with the published literature, 100% completeness was not reached for all data elements. This is probably, to some extent, an inevitable consequence of necessary flexibility in any reporting system. In many instances, it will not be reasonable to entirely block validation if a pathologist is unable to give a response to a certain data element for a specific case. Only a single optional data element (“Additional pathologic findings”) for each cancer protocol was reported less frequently with the synoptic format than in the narrative reports, possibly because the template contained a note that only relevant findings should be reported, which may have prompted pathologists not to report non-neoplastic findings lacking clinical significance. Parenthetically, synoptic format might also be used to improve consistency and completeness of reporting of clincally relevant non-neoplastic findings, even though this would be beyond the scope of the present study. Conversely, > 95% completeness was already achieved with narrative reports for data elements covered by the previously existing dictation template for colon cancer or the TNM template used for both cancer types.

Limitations of our study arise from the fact that we assessed completeness of reports only for two protocols for relatively common cancer types reported in majority by non-subspecialized pathologists. Therefore, the increase in completeness might have been smaller for cases handled by subspecialized pathologists. One study, however, analyzing the impact of synoptic reporting on reports for malignant melanoma found increased completeness irrespective of subspecialization [11]. On the other hand, the rate of completeness was already relatively high for narrative reports in our institution, which appears to be due to the previously existing dictation templates, which already included many of the required data elements. Finally, the transfer of our findings to other settings may be limited by the fact that our specific approach of template-based reporting was tailored to our laboratory information system. Such differences between pathology departments might result in different actual figures for completeness of narrative and synoptic reports, respectively. The general finding, however, i.e., high levels of completeness with template-based synoptic reports, should be largely translatable to other institutions as most laboratory information systems would be expected to feature at least basic functionalities for utilization of templates.

While we did not attempt to quantitatively assess pathologists’ adherence to specific wordings, we found that, generally, the terminology used in synoptic reports was very consistent with the one suggested by our translations of the CAP protocols. On the other hand, we occasionally observed minor deviations such as typographical errors, which might have been avoided with a well-designed database solution.

Despite its benefits being widely acknowledged, synoptic reporting is arguably significantly underutilized. A mixture of psychological and technological factors contributes to this phenomenon. Specifically, the setup of a database structure for synoptic reporting on a single-institutional level and the continuous maintenance of a broad variety of protocols imposes a significant burden on a department of pathology. Furthermore, frustration with workflow issues associated with less than optimal database solutions may interfere significantly with the acceptance of synoptic reporting by pathologists [12, 13].

These considerations prompted us to implement synoptic reporting at our institution through a transitory phase in which we introduced synoptic protocols for a variety of cancer types while deferring an underlying database structure. For this purpose, we relied on the inbuilt autotext function of our laboratory information system. Thereby, reports are created, which are essentially equivalent to synoptic reports generated from a database for the readers, i.e., surgeons, radiation and medical oncologists, or pathologists (e.g., for the presentation in a multidisciplinary tumor conference). Furthermore, the anticipated multiple minor changes required in the early phase of practical implementation of each protocol were easy to make in this system. We reasoned that this approach would facilitate getting started with synoptic reporting and that the experience gained from this phase would then improve a subsequent database solution and smoothen the transition.

The easy adaptability of our template-based system enabled us to rapidly implement more than 20 surgical and biomarker protocols. It was particularly useful in the early phase when multiple minor changes with regard to wording or formatting could be made very easily and fast in comparison to often lengthy software development cycles. On the other hand, our approach should not be considered a definite solution for several reasons: (1) An underlying database structure is indispensable for fully automated data retrieval and data exchange; (2) the template-based system faces limitations when it comes to complex conditional data elements (e.g., extranodal extension being only relevant when a lymph node metastasis is present); (3) background information on specific data elements or responses cannot be easily highlighted to the pathologist with the template-based format; (4) continuous updates of the protocols result in a significant workload.

We, nevertheless, believe that the experiences from this transitory phase of template-based synoptic reporting provided critical input for the ongoing implementation of a database for synoptic reporting on a national level [7]. This experience relates to a variety of issues, including consistent translation of English protocols, policies regarding the inclusion of additional data elements, the handling of optional or conditional data elements, and formatting of the reports. All of these issues can be challenging to address individually but may become disproportionately more complicated when interfering with information technology-related issues.

In conclusion, we have shown that template-based synoptic reporting without underlying database structure may be a useful transitional step on the path toward higher levels of data structuring, especially for initiatives within a single institution. From a longer-term perspective, however, the pathology community needs to find ways to provide systems, which reduce the burden of local implementation of synoptic reporting with regard to both consistent terminology across languages and a framework for data structure and data exchange with local laboratory information systems. Only, thereby, synoptic reporting will be able to unfold its full potential for cancer care and cancer research on a global level.

## Data Availability

The full templates are available from the corresponding author at request.
